# Wearable Hybrid Strain–Myoelectric Sensing System for Machine‐Learning‐Assisted Sarcopenia Screening

**DOI:** 10.1002/smsc.202500582

**Published:** 2026-03-28

**Authors:** Ke Wang, Guanbo Min, Tingyu Wang, Chengyu Li, En Zhao, Kun Xu, Yuer Liang, Zhiwei Wang, Qianmei Sun, Zhiyi Gao, Jing Chang, Wei Tang

**Affiliations:** ^1^ Beijing Institute of Nanoenergy and Nanosystems Chinese Academy of Sciences Beijing China; ^2^ School of Nanoscience and Technology University of Chinese Academy of Sciences Beijing China; ^3^ School of Fashion and Textiles The Hong Kong Polytechnic University Kowloon China; ^4^ Department of Emergency Medicine Beijing Chaoyang Hospital Capital Medical University Beijing China; ^5^ Department of Orthopaedics Beijing Chaoyang Hospital Capital Medical University Beijing China; ^6^ Department of Nephrology Beijing Chaoyang Hospital Capital Medical University Beijing China; ^7^ CAS Key Laboratory of Magnetic Materials and Devices Ningbo Institute of Materials Technology and Engineering Chinese Academy of Sciences Ningbo China; ^8^ Department of Internal Medicine Beijing Chaoyang Hospital Capital Medical University Beijing China

**Keywords:** convolutional neural network–long short‐term memory, interpretability analysis, muscle fiber, sarcopenia, wearable electronics

## Abstract

The early screening of sarcopenia represents a critical clinical need amid the accelerating global aging population. Current diagnostic methods, relying on bioelectrical impedance analysis (BIA), handgrip strength testing, and other clinical examinations, depend on costly medical equipment and struggle to concurrently assess both muscle mass and strength. Herein, we propose a Wearable Sarcopenia Assessment System (WSAS), which employs an integrated hybrid surface electromyography (sEMG)‐piezoelectric strain sensing platform to synchronously capture electrophysiological signals and mechanical deformation signals during muscle contraction in handgrip tests (signal‐to‐noise ratio: 34.32 dB), and incorporates a CNN‐LSTM deep learning framework. This model was trained using nine physiologically relevant features (including root mean square (RMS), mean absolute value (MAV), and integrated EMG (iEMG)) extracted through feature engineering as prior knowledge. Validated in a cohort of 75 elderly participants, the proposed system achieved a screening accuracy of 99.85% with an area under the curve (AUC) of 0.97. Shapley additive explanations (SHAP)‐based interpretability analysis further revealed that WSAS captures neuromuscular alterations associated with sarcopenia, including type II‐to‐type I muscle fiber transition and neuromuscular junction remodeling. These results demonstrate the potential of WSAS as a portable, low‐cost, and radiation‐free platform for early‐stage sarcopenia screening.

## Graphical abstract

Hybrid electrophysiological–mechanical sensing meets interpretable deep learning in a wearable platform for early sarcopenia screening. The Wearable Sarcopenia Assessment System synchronously captures surface electromyography and piezoelectric strain signals and leverages physiologically guided Convolutional Neural Network–Long Short‐Term Memory analysis to reveal neuromuscular alterations, offering a portable, low‐cost, and radiation‐free solution for future home‐based monitoring and precision aging healthcare.

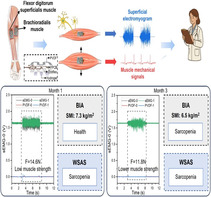

## 1 Introduction

The global population aged 60 years and older is projected to double within the next three decades, a demographic shift that is expected to place increasing pressure on healthcare systems worldwide [1]. Sarcopenia, a condition prevalent among the elderly, has garnered considerable attention due to its association with increased risks of falls, fractures, and complications, including metabolic disorders, dysphagia, and impaired cardiopulmonary function [2, 3]. It is a progressive musculoskeletal disorder characterized by accelerated loss of muscle mass, strength, and function [4]. Epidemiological studies estimate a global prevalence of approximately 10%–15% in both developed and developing countries [5, 6]. The early symptoms of sarcopenia can be effectively mitigated through targeted rehabilitation exercises and nutritional interventions. Importantly, underscoring the need for accurate and rapid early identification strategies [7, 8, 9].

Clinically, sarcopenia is diagnosed in individuals presenting with low muscle mass concomitant with diminished muscle strength [10, 11]. Computed tomography (CT) and dual‐energy X‐ray absorptiometry (DXA) are considered reference standards for assessing muscle mass; however, their widespread adoption is hindered by concerns over radiation exposure and high costs [12]. BIA provides a more accessible alternative by estimating body composition through tissue conductivity, yet its accuracy is affected by physiological factors such as hydration status and skin temperature [13, 14]. Consequently, comprehensive assessment strategies that integrate measurements of muscle mass, strength, and functional performance have gained increasing interest. Age‐related reductions in muscle fiber number alter muscle cross‐sectional area, changes that may be detectable using wearable sensing technologies during dynamic contraction [15, 16, 17]. For example, Han et al. reported a BaTiO_3_‐based piezoelectric strain sensor achieving over 90% accuracy in sarcopenia assessment [18]. Meanwhile, early manifestations of sarcopenia often involve neuromuscular impairment, suggesting that sEMG features obtained during handgrip testing may capture neuromuscular junction (NMJ) characteristics [19, 20, 21]. Li et al., for instance, achieved a screening accuracy of 73% using sEMG signals [22]. However, strain signals are susceptible to motion artifacts, reducing reliability [23], whereas sEMG alone may struggle to distinguish whether strength decline arises from reduced muscle fiber quantity or impaired neural drive [24]. These limitations highlight the need for sensing approaches capable of simultaneously assessing both mechanical and electrophysiological aspects of muscle function [25, 26, 27, 28, 29]. Integrating sEMG with strain sensing therefore represents a promising pathway toward more comprehensive and clinically meaningful sarcopenia screening [30, 31].

Here, we present a Wearable Sarcopenia Assessment System (WSAS) based on a hybrid sensing architecture that combines sEMG and piezoelectric strain sensors to synchronously acquire coupled electrophysiological and mechanical signals during muscle contraction (signal‐to‐noise ratio: 34.32 dB). In this pilot feasibility study, 75 elderly participants aged 60 years or older were recruited to evaluate the system. The WSAS device was affixed to the brachioradialis (BR) and flexor digitorum superficialis (FDS) muscles during standardized handgrip tests. Following data acquisition, nine engineered signal features were extracted and incorporated as prior knowledge into a Convolutional Neural Network–Long Short‐Term Memory (CNN‐LSTM) model for condition assessment [32, 33], achieving an accuracy of 99.85%. Furthermore, SHAP‐based interpretability analysis was employed to relate model outputs to physiologically relevant signal patterns potentially associated with muscle fiber transitions and neuromuscular remodeling. This design enhances both interpretability and feasibility, positioning WSAS as a promising wearable platform for early‐stage sarcopenia screening.

## 2 Results

### 2.1 Hybrid Sensor Characteristics

**FIGURE 1:**
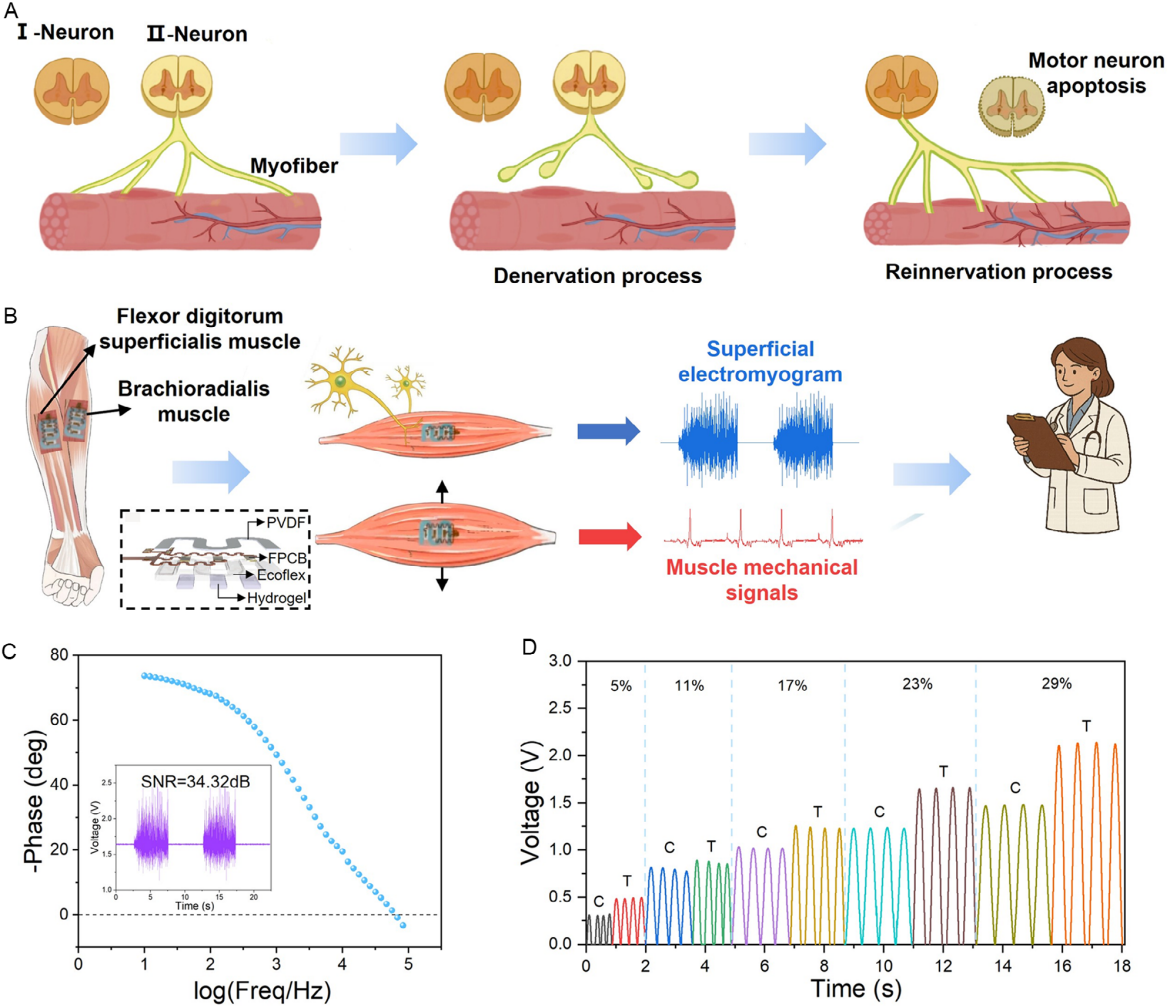
Schematic of the WSAS system for sarcopenia diagnosis. (A) Schematic illustration of sarcopenia pathogenesis. Depicts the process from denervation of type II muscle fibers to their reinnervation and subsequent transformation into type I fibers. (B) Schematic diagram of the sarcopenia diagnosis process using the WSAS system (The illustration is an exploded view of the coupled sensor). Integrated sensors adhered to the BR and FDS muscles synchronously acquire sEMG and mechanical signals generated during grip strength testing. (C) Impedance curve of the hydrogel. The inset shows its average SNR of 34.32 dB when used as an sEMG electrode. (D) Characterization of the serpentine piezoelectric sensor's tensile and compressive properties.

Muscle fiber type transformation is widely recognized as an important mechanism associated with the progression of sarcopenia [4]. During aging, excessive accumulation of reactive oxygen species (ROS) triggers oxidative damage, causing motoneurons that innervate fast muscle fibers (type II) to undergo apoptosis more readily [34, 35], which may contribute to preferential denervation of type II fibers. Subsequently, these denervated fibers are reinnervated by motoneurons that normally innervate slow muscle fibers (type I) [25, 36]. The lowfrequency stimulation applied during reinnervation activates peroxisome proliferator‐activated receptor‐γ coactivator‐1α (PGC‐1α) [37, 38]. PGC‐1α upregulates mitochondrial transcription factor A (TFAM) to drive mitochondrial biogenesis and elevates vascular endothelial growth factor (VEGF) levels to increase capillary density [39], thereby shortening the oxygen diffusion distance within muscle fibers, which favors the endurance contractions of type I fibers [40]. Meanwhile, denervation together with reduced physical activity disrupts the Akt–mTOR signaling pathway [41]. Specifically, chronic overactivation of mTORC1 suppresses effective synthesis of myofibrillar proteins such as myosin, whereas Akt inactivation releases the transcription factor FoxO3a [42], which upregulates protein catabolism via the E3 ubiquitin ligases MuRF‐1 and Atrogin‐1 [43]. The combined effects of inhibited synthesis and enhanced degradation cause a net protein loss, ultimately reducing the cross‐sectional area of muscle fibers. In addition, as a compensatory mechanism, the length and number of axonal branches at the NMJ increase, the total number of motor units (MU) decreases, and the remaining MUs enlarge in size (as shown in Figure 1A) [44], indicating that the body attempts to maintain neuromuscular transmission efficiency by expanding the innervation territory of individual MUs.

Figure 1B illustrates the hybrid sensing principle of the WSAS system, which integrates a polyvinylidene fluoride (PVDF)‐based strain sensor with hydrogel‐based sEMG electrodes (Figure S1). Additional device details are provided in our previous work [45]. After positioning the system over the brachioradialis (BR) and flexor digitorum superficialis (FDS) muscles, participants performed standardized handgrip tasks. Activation of motor neurons generates motor unit action potentials (MUAPs) that propagate along axons toward the NMJ and initiate excitation of muscle fibers. The hydrogel electrodes record potential variations reflecting MU recruitment status and neuromuscular activation patterns, forming the sEMG signal. Simultaneously, the integrated PVDF strain sensor (thickness: 100 μm) captures mechanical deformation associated with muscle contraction. These synchronized electrophysiological and mechanical signals are transmitted to a computer for machine‐learning‐assisted assessment.

The hydrogel electrodes were fabricated using a stretchable ionic hydrogel based on a polyrotaxane structure, exhibiting high conductivity (6.58 S m^−1^), as shown in Figure 1C. The inset of Figure 1C presents an average sEMG signal‐to‐noise ratio (SNR) of 34.32 dB across ten repeated measurements. Additional EMG data under varying grip strengths are provided in Figure S2. To enhance mechanical compliance, a serpentine geometry was employed for the PVDF strain sensor. Experimental characterization demonstrated that the device could detect strains as low as 5% (corresponding to an approximate stretch distance of 1 mm) under both tensile and compressive conditions. Within a strain range of 5%–29% (corresponding to stretch distances of 1 mm to 5.6 mm), the output exhibited a strong linear relationship with applied strain (Figure 1D). This performance supports reliable capture of muscle contraction dynamics and provides a foundation for synchronized multimodal sensing analysis.

### 2.2 Experimental Protocol and Signal Processing

**FIGURE 2:**
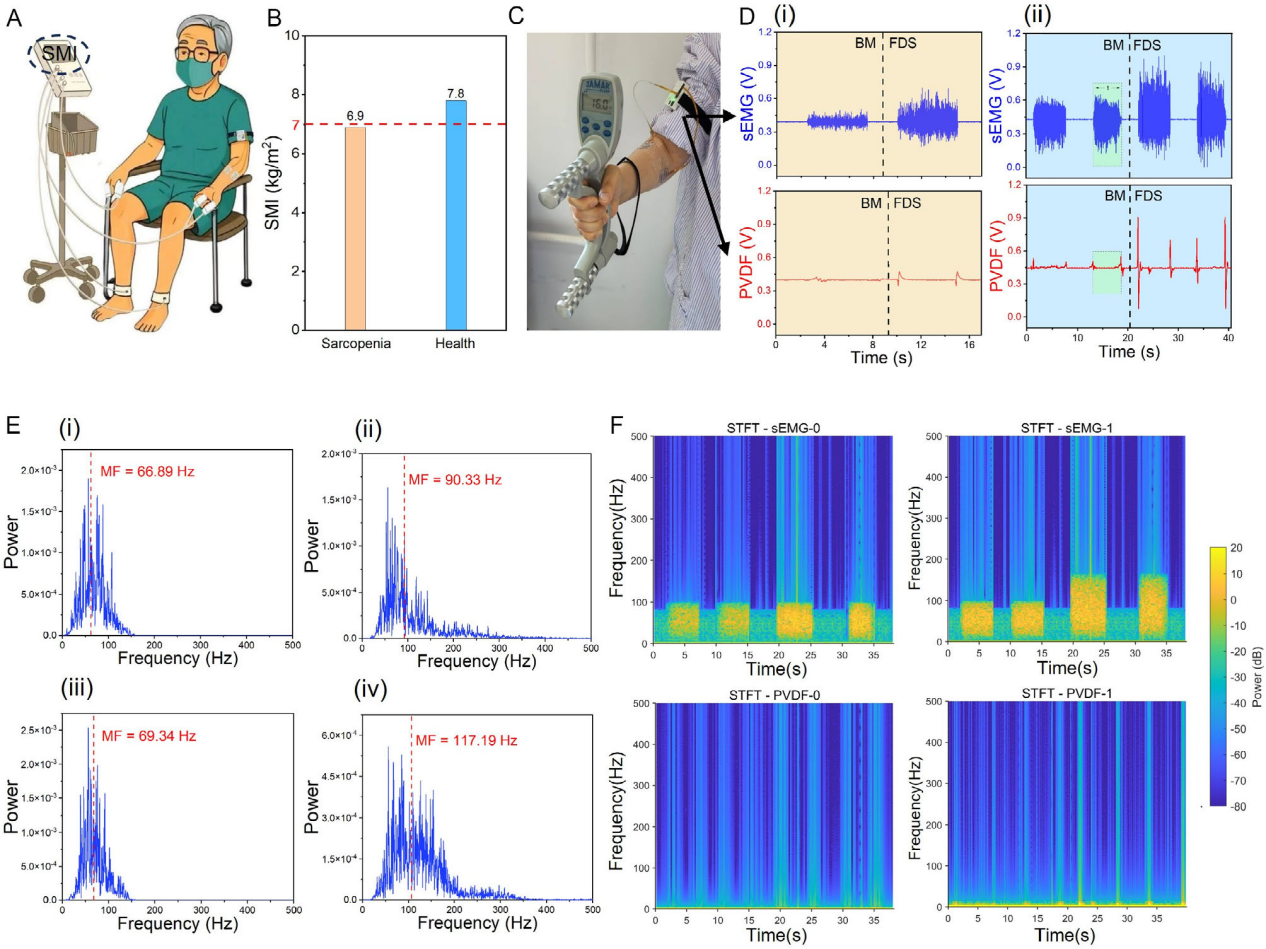
Testing procedure and signal characterization. (A) Participants were grouped according to the gold‐standard SMI measured with a body‐composition analyzer. (B) SMI values of a male subject before and after he was diagnosed with sarcopenia. (C) Photograph of the patient wearing the WSAS during a grip‐strength test. (D) Normalized sEMG signals and normalized mechanical signals recorded before and after diagnosis; (i) denotes post‐diagnosis, and (ii) denotes pre‐diagnosis. (E) Computed median frequency of the sEMG signals; panels (i) and (iii) and panels (ii) and (iv) correspond to the BM and FDS muscles in Figure D(i) and Figure D(ii), respectively. (F) Spectrogram obtained by concatenating the patient's normalized signals before and after sarcopenia diagnosis and applying a STFT.

Data were acquired following a standardized handgrip testing protocol. Prior to the grip strength assessment, each participant underwent bioelectrical impedance analysis (BIA) (Figure 2A), and the corresponding results are summarized in Figure 2B. To improve electrode–skin contact stability, the stratum corneum of the upper arm was gently cleaned with an alcohol swab before electrode placement. Two pairs of hydrogel electrodes were then positioned over the brachioradialis (BR) and flexor digitorum superficialis (FDS) muscles and secured using medical adhesive tape. Participants were instructed to sit upright with the tested elbow flexed at 90° while holding a standard dynamometer (JAMAR) with the dominant hand; the non‐tested arm remained relaxed at the side (Figure 2C). Each participant performed three maximal voluntary contractions (MVCs), each lasting 5 s with 4–6 s rest intervals. The mean force across the three trials was defined as the participant's grip strength.

During testing, synchronized electrophysiological–mechanical signals were recorded at a sampling rate of 1000 Hz using an eight‐channel STM32‐based acquisition system and wirelessly transmitted to a computer workstation via Wi‐Fi (Figures S3 and S4). A custom LabVIEW program enabled real‐time data visualization, synchronization, and storage. Figure 2D(i,ii) present representative signal patterns from one participant recorded 3 months before and after a clinical diagnosis of sarcopenia, where panel (i) corresponds to the post‐diagnosis state and panel (ii) to the pre‐diagnosis state (raw signals prior to normalization are shown in Figure S5a,b). Compared with the pre‐diagnosis condition, both sEMG and piezoelectric signals exhibited reduced amplitudes following disease progression, reflecting diminished neuromuscular activation and mechanical contraction capacity.

To further explore neuromuscular characteristics, the median frequency (MF) of sEMG within time window *t* was analyzed (Figure 2E). Panels (i) and (iii) represent MF values of BR and FDS after diagnosis, whereas panels (ii) and (iv) correspond to measurements obtained prior to diagnosis. MF values decreased from 90.33 Hz and 117.19 Hz to 66.89 Hz and 69.34 Hz, indicating a pronounced shift toward lower‐frequency components. As MF is commonly associated with muscle fiber composition and fatigue characteristics, these changes suggest alterations in neuromuscular activation patterns consistent with sarcopenia‐related adaptations. Short‐time Fourier transform (STFT) analysis of concatenated and normalized signals (Figure 2F) further revealed that the sarcopenia group exhibited stronger energy concentration in lower‐frequency bands and fewer high‐frequency components compared with the healthy group. A similar trend was observed in the piezoelectric strain signals, supporting the consistency between electrophysiological and mechanical sensing modalities.

Notably, the WSAS platform demonstrated sensitivity to weak muscle activity signals characteristic of sarcopenia. Distinct feature variations observed within the same participant across disease progression provided an informative basis for subsequent deep learning model development.

### 2.3 CNN‐LSTM‐Based Prior Knowledge Classification Model

**FIGURE 3:**
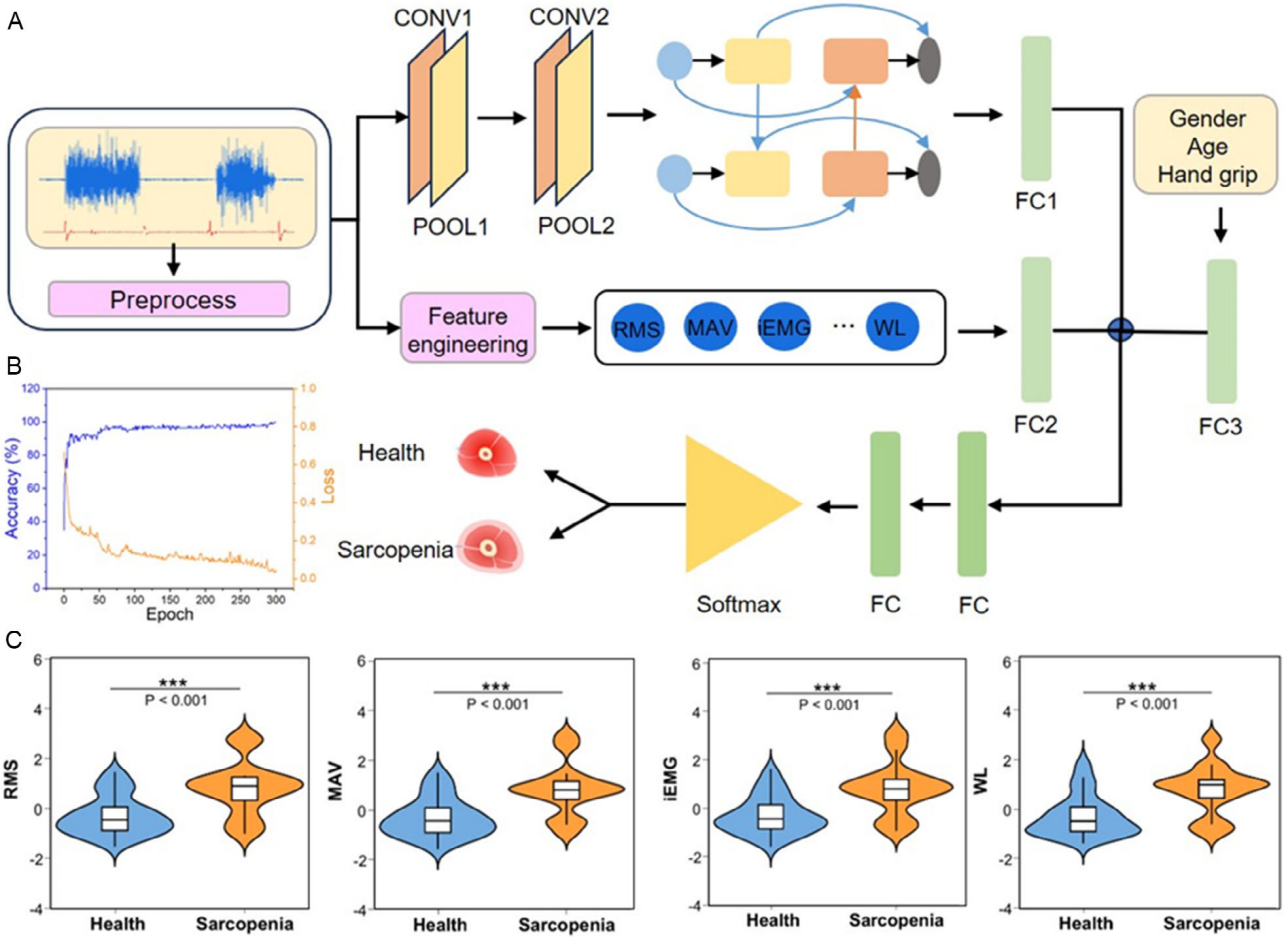
CNN–LSTM‐based sarcopenia classification. (A) Architecture of the CNN–LSTM network, comprising data preprocessing, CNN–LSTM, feature‐engineering and classification modules. (B) Training accuracy and loss curves of the CNN–LSTM model. (C) Violin plots of four pathological features extracted by feature engineering for both sarcopenia and healthy groups: RMS, MAV, iEMG, and CWT_power.

Building upon the multimodal characteristics of electrophysiological and mechanical signals, this study employed a hybrid deep learning architecture that integrates a convolutional neural network with a long short‐term memory network (CNN–LSTM). Using the multimodal signals acquired from the BR muscle as an illustrative example (Figure 3A), the network consists of three core modules: a Spatial Feature Extraction Module, a Feature Engineering Module, and a Fusion Classification Module. For spatial feature extraction, the CNN adopts a two‐layer convolutional architecture to hierarchically learn representations from the four‐channel time‐series sEMG–piezoelectric inputs using ReLU activation. The resulting feature maps are downsampled through pooling operations, flattened into one‐dimensional vectors, and subsequently fed into an LSTM layer with 64 hidden units to model temporal dependencies embedded in the sequential data. To further enhance model robustness and clinical relevance, two auxiliary inputs were incorporated. First, nine handcrafted sEMG‐derived features extracted by the Feature Engineering Module were introduced as prior physiological knowledge. Second, subject‐level variables—including gender, age, and grip strength—were included to mimic clinically relevant indicators commonly used in body composition assessment. These three feature streams were aligned through convolution‐based dimensional mapping and jointly integrated into the network for end‐to‐end training. Finally, the Fusion Classification Module outputs the sarcopenia classification result via a Softmax classifier. As shown in Figure 3B, the training process demonstrates stable convergence, with the CNN–LSTM model achieving a peak accuracy of 99.85% and a loss value of approximately 0.001.

To improve model interpretability and strengthen the connection with clinical pathology, feature engineering was conducted on valid signal segments within the analysis window. Nine physiologically meaningful features were extracted from both time‐domain and time–frequency representations. Six classical Hudgins time‐domain features were included: RMS, MAV, iEMG, Waveform Length (WL), Zero Crossings (ZC), and Slope Sign Changes (SSC). In addition, three time–frequency features were derived from Continuous Wavelet Transform (CWT) coefficients: Absolute Power (CWT_power), Kurtosis (CWT_kurtosis), and Wavelet Entropy (WE). All features were normalized to the mean value of the three MVC trials to reduce inter‐individual variability. Figure 3C presents violin plots comparing the distributions of four representative features—RMS, MAV, iEMG, and CWT_power—between the sarcopenia and healthy control groups, revealing statistically significant differences (*p* < 0.001). Violin plots of the remaining five features (ZC, SSC, WL, CWT_kurtosis, and WE) are shown in Figure S6, where WE, CWT_kurtosis, and ZC did not exhibit significant intergroup differences. Notably, the sarcopenia group displayed higher mean values of RMS, MAV, iEMG, and WL compared with the healthy group. RMS reflects the root–mean‐square amplitude of sEMG activity, whereas MAV represents the overall signal magnitude; both increase with elevated muscle fatigue. These findings suggest that sarcopenic individuals require greater neuromuscular activation to sustain maximal grip force. iEMG quantifies the cumulative electrical activity during contraction and correlates with motor unit recruitment intensity, while WL captures waveform complexity. The observed increases in iEMG and WL indicate enhanced compensatory recruitment of motor units during MVC tasks, which is consistent with denervation‐driven remodeling mechanisms discussed earlier.

### 2.4 Model Evaluation

**FIGURE 4:**
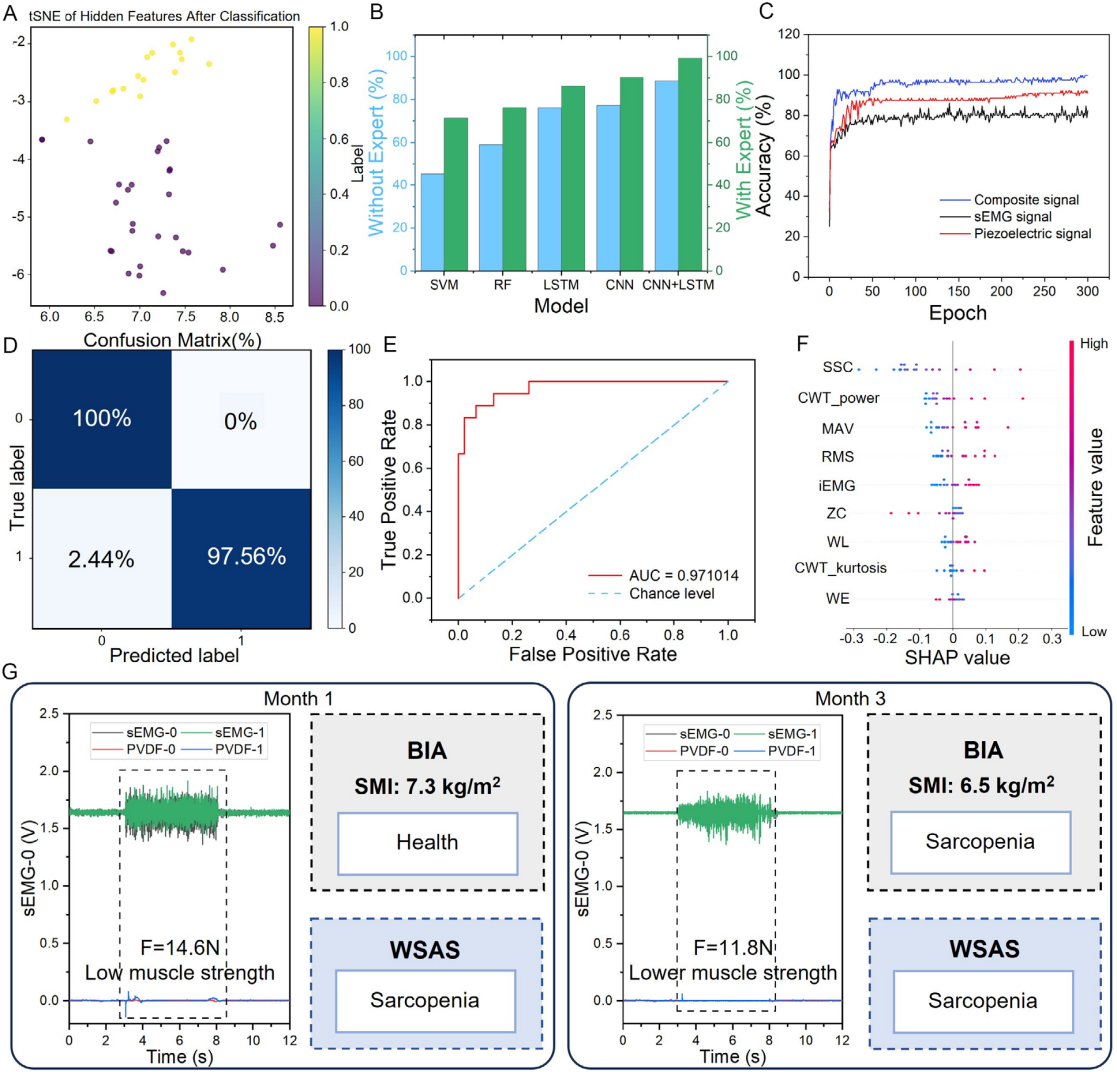
CNN‐LSTM model evaluation. (A) t‐SNE visualization of the features after the CNN‐LSTM module. (B) Bar chart comparing the classification accuracy of CNN‐LSTM with other deeplearning models (SVM, RF, LSTM, and CNN) for sarcopenia detection. CNN‐LSTM outperforms all other models, with an average accuracy of about 99.34%. (C) Relationship between training epochs and accuracy of the CNN‐LSTM model using sEMG, piezoelectric, and coupled signals (sEMG–piezoelectric coupling). (D) Confusionmatrix probabilities for sarcopenia classification based on CNN‐LSTM, showing a maximum accuracy of approximately 99.85%. (E) ROC‐AUC results of the CNN‐LSTM model. The blue dashed line indicates chance level (i.e., random guessing), and the red solid line shows the model's ROC curve. (F) SHAP summary plot. Each point represents the SHAP value of a feature for one subject. The vertical axis lists the features, and the horizontal axis shows the SHAP values; redder colors indicate higher feature values. (G) Schematic application of the WSAS: a male patient had an SMI of 7.3 kg/m^2^ measured by BIA in the first month, which is within the healthy range, whereas the WSAS system diagnosed sarcopenia. In the third month, the SMI dropped to 6.5 kg/m^2^, indicating sarcopenia, and the WSAS diagnosis agreed with the BIA result.

To evaluate the effectiveness of the proposed CNN–LSTM hybrid architecture in feature representation and classification, t‐distributed stochastic neighbor embedding (t‐SNE) was applied to visualize the high‐dimensional feature space. As shown in Figure 4A, multimodal signal features extracted from the BR muscle formed well‐separated clusters in the two‐dimensional projection, where samples from the healthy control group and the sarcopenia group exhibited clear spatial segregation. This visualization further supports the superior representation capability of the hybrid architecture in capturing discriminative multimodal patterns. In the comparative experiments, the classification performance of five representative machine‐learning models was systematically evaluated (Figure 4B), including Support Vector Machine (SVM), Random Forest (RF), CNN, LSTM, and the proposed CNN–LSTM framework. Results indicate that the CNN–LSTM model incorporating prior physiological knowledge consistently outperformed all baseline models across evaluation metrics, achieving an average classification accuracy of 99.85% (Figure 4D) with only one misclassified sample.

To further investigate the synergistic contribution of multimodal sensing, three signal configureurations were compared for sarcopenia classification (Figure 4C): sEMG signals alone, piezoelectric strain signals alone, and their combined input. Ten‐fold cross‐validation results demonstrated that the multimodal fusion strategy achieved the highest average accuracy (≈99.85%), outperforming both single‐modality inputs. This improvement suggests that electrophysiological activity and mechanical deformation provide complementary information that jointly reflects the neuromuscular and biomechanical alterations associated with sarcopenia. The discriminative performance of the model was further assessed using receiver operating characteristic (ROC) analysis. As shown in Figure 4E, the area under the curve (AUC) reached 0.97, with the ROC curve approaching the upper‐left corner, indicating excellent binary classification capability. SHAP‐based feature attribution analysis (Figure 4F) revealed that RMS, MAV, iEMG, and CWT_power were among the most influential contributors to model decisions, which is consistent with the fatigue‐related and compensatory neuromuscular patterns discussed in earlier sections.

Figure 4G presents a representative clinical application scenario of the WSAS system. A male participant initially presented with a skeletal muscle index (SMI) of 7.3 kg m^−2^ measured by BIA, which did not meet the diagnostic threshold for sarcopenia (cut‐off: 7.0 kg m^−2^) and was therefore categorized as healthy according to conventional criteria. However, the WSAS system classified the participant into the sarcopenia group, accompanied by a reduced grip strength of 14.6 N, suggesting early electrophysiological and mechanical abnormalities. At a 3‐month follow‐up assessment, the participant's SMI decreased to 6.5 kg m^−2^ and grip strength further declined to 11.8 N, aligning with the earlier prediction generated by the machine‐learning model. These observations highlight the potential of the WSAS platform for early‐stage screening and timely intervention in sarcopenia management.

## 3 Conclusion

The WSAS platform developed in this study integrates sEMG and PVDF‐based sensing technologies, enabling synchronous acquisition of electrophysiological and mechanical signals during the muscle excitation–contraction process. Compared with conventional diagnostic approaches such as DXA and BIA, the proposed system offers several key advantages, including radiation‐free operation, low cost, and portability, highlighting its potential for wearable and point‐of‐care applications. By leveraging the complementary characteristics of sEMG and piezoelectric strain signals, together with physiologically informed features introduced as prior knowledge, the CNN–LSTM framework achieved highly sensitive and specific sarcopenia screening (99.85% accuracy; AUC = 0.97). The superior ability of the hybrid architecture to capture temporal and multidimensional signal patterns demonstrates the promise of deep learning for interpreting complex neuromuscular alterations. Moreover, SHAP‐based interpretability analysis identified clinically meaningful electrophysiological biomarkers associated with sarcopenia, providing a physiological rationale for model decisions and offering new perspectives for early biomarker discovery.

Despite these encouraging results, several limitations should be acknowledged. First, participants were recruited from a single medical center with a relatively limited cohort size (*n* = 75); future multicenter studies with larger populations are required to validate generalizability. Second, the combined sEMG–PVDF signal has not yet been established as a clinical gold standard for sarcopenia diagnosis. In addition, the current framework focuses on binary screening rather than severity grading. Future work will therefore explore expanded datasets, multimodal assessments of lower‐limb function, and real‐time embedded deployment of the WSAS system. With further development, the proposed platform may support continuous home‐based monitoring, personalized rehabilitation guidance, and early risk stratification in aging populations, advancing the translation of wearable neuromuscular sensing technologies toward practical clinical use.

## 4 Experimental Methods

### 4.1 Participants

Consecutive recruitment of adults aged over 60 years was conducted in Beijing for sarcopenia screening. To ensure research quality, the exclusion criteria for this study were: (1) having severe acute conditions (e.g., acute myocardial infarction, stroke within 2 weeks of onset, severe infection with high fever); (2) severe cognitive impairment or consciousness disturbance preventing cooperation with measurements; (3) severe motor dysfunction precluding completion of muscle strength assessment; (4) acute limb injury or severe pain interfering with testing; (5) significant edema, ascites, or skin lesions/infections affecting measurement accuracy; and (6) active malignancy. Ultimately, 75 eligible participants were enrolled (Table S1) and completed all study procedures.

This work was conducted in accordance with the World Medical Association's Declaration of Helsinki (2000). The study protocol received approval from the Medical and Pharmacy Ethics Committee of Beijing Chaoyang Hospital, Capital Medical University (Approval No. 2022‐K‐531), and written informed consent was obtained from all participants.

### 4.2 Sarcopenia Screening

The 2019 Asian Working Group for Sarcopenia (AWGS) diagnostic criteria were used to identify participants with sarcopenia. First, handgrip strength assessment was performed for each participant using a standard electronic dynamometer (JAMAR, USA). Handgrip strength below 28 kg for men or below 18 kg for women was classified as low muscle strength. Subsequently, muscle mass was evaluated via bioelectrical impedance analysis (BIA) using the InBody S10 device (Seoul, Korea), with the skeletal muscle mass index (SMI) calculated. An SMI below 7.0 kg/m^2^ for men or below 5.7 kg/m^2^ for women was defined as low muscle mass. Finally, physical performance was assessed by measuring the time required to complete five chair stands; a time ≥12 s was considered indicative of poor physical performance. Participants were diagnosed with sarcopenia and assigned to the sarcopenia group if they exhibited low muscle mass plus either low muscle strength, poor physical performance, or both. Participants demonstrating normal values for muscle mass, muscle strength, and physical performance were assigned to the healthy control group.

### 4.3 Preparation of Hydrogel Electrodes

The hydrogel was synthesized by introducing acrylamide (AM) and N, N’‐methylenebisacrylamide (BIS) as dual crosslinkers, the conductive polymer poly(3,4‐ethylenedioxythiophene): poly(styrenesulfonate) (PEDOT: PSS), and a lithium chloride (LiCl) ionic solution, with tetramethylethylenediamine (TMEDA) serving as the polymerization catalyst. The specific procedure was as follows: 6 g of AM solid particles and 5 g of LiCl particles were dissolved in 9.5 mL of deionized water under continuous magnetic stirring at 300 rpm until a homogeneous aqueous solution was obtained. Subsequently, 0.005 g of BIS and 1.5 mL of PEDOT: PSS solution (conductivity: 700–800 S cm^−1^) were added to the solution, followed by the addition of 0.05 g of ammonium persulfate (APS). The mixture was stirred at room temperature for 15 min to ensure complete dissolution of the added materials. The resulting solution was poured into a pre‐fabricated glass mold, and 10 µL of TMEDA catalyst was added dropwise under uniform stirring. This initiated a spontaneous self‐polymerization exothermic reaction, leading to the formation of a polyrotaxane structure and the generation of a viscous ionic hydrogel.

### 4.4 Feature engineering

(1) Root Mean Square (RMS)

RMS reflects the effective value of the electrical discharge. Its magnitude is determined by the variation in sEMG amplitude and is generally associated with motor unit recruitment and the synchronization of firing patterns. Its peak value reflects the amplitude or contraction intensity, primarily governed by the underlying relationship between muscle loading factors and the muscle's intrinsic physiological and biochemical processes.

(1) $$RMS=\sqrt{\frac{1}{N}\sum_{I=1}^{N}x_{i}^{2}}$$

(2) Integrated Electromyography (iEMG)

iEMG represents the cumulative area under the electromyographic signal curve per unit time. It reflects the total discharge of motor units active within the muscle over a specified period. Under the condition of constant time duration, the magnitude of iEMG indicates both the number of motor units recruited and the discharge level of each motor unit, thereby characterizing the muscle's contraction properties within a unit time frame.

(2) $$iEMG=\int_{t_{1}}^{t_{2}}|X\left(t\right)|dt$$

(3) Mean Absolute Value (MAV)

MAV is the average of the instantaneous amplitude of the electromyogram over a period of time. Its variation mainly reflects the number of motor units activated during muscle activity, the types of motor units involved in the activity, and their degree of synchronization, which is related to the central control function under different muscle load intensity conditions and can be used to evaluate muscle endurance.

(3) $$MAV=\frac{1}{N}\sum_{I=1}^{N}x(i)$$

(4) Waveform Length (WL)

This characteristic refers to the cumulative sum of the WLs of the sEMG signal within a certain time window, indicating the complexity of the surface electromyographic signal.

(4) $$WL=\sum_{I=1}^{N}|x_{i+1}-x_{i}|$$

## Supporting Information

Additional supporting information can be found online in the Supporting Information section. **Supporting Fig. S1:** Shown are the detailed components and photographs of the wearable sensor for sarcopenia assessment. (a) The detailed components include FPCB1, FPCB2, a 3D mold, a serpentine piezoelectric PVDF film, and a hydrogel layer. (b) These components are subsequently integrated into a sensor that adheres to the patient's muscle and can simultaneously acquire electromyographic and piezoelectric signals. **Supporting Fig. S2:** Schematic of electromyographic signals recorded during a standard gripstrength test at various force levels (5 N, 10 N, 15 N, 20 N, and 30 N). **Supporting Fig. S3:** Schematic of the WSAS signalprocessing flow. The piezoelectric and electromyographic signals are amplified by their respective amplifier circuits, undergo analogtodigital conversion and signal processing under the control of the main controller, and are ultimately transmitted wirelessly to the terminal via the Wi‐Fi protocol. **Supporting Fig. S4:** Photograph of the integrated signal‐processing circuit, showing the signal amplification, signal conversion, main control unit, and wireless transmission module. **Supporting Fig. S5:** Unnormalized data of the patient before and after being diagnosed with sarcopenia. (a) Unnormalized data corresponding to Fig. 2D(i); (b) Unnormalized data corresponding to Fig. 2D(ii). **Supporting Fig. S6:** Violinplot matrix illustrating the differences in CWT_power, WE, CWT_kurtosis, SSC, and ZC. CWT_power differed significantly between the sarcopenia patients and the healthy group, SSC also showed a difference, whereas WE, CWT_kurtosis, and ZC did not exhibit clear differences in this study. **Supporting Table S1:** Descriptive statistics of all participants represented as mean (±SD).

## Author Contributions

**Ke Wang:** conceptualization (lead), data curation (lead), formal analysis (lead), investigation (lead), methodology (lead), software (lead), visualization (lead), writing – original draft (lead). **Guanbo Min:** formal analysis (equal), funding acquisition (equal), methodology (equal), writing – original draft (equal). **Tingyu Wang:** formal analysis (equal), software (supporting), visualization (supporting). **Chengyu Li:** methodology (supporting), software (supporting). **En Zhao:** data curation (supporting), software (supporting). **Kun Xu:** software (supporting). **Yuer Liang:** data curation (supporting). **Zhiwei Wang:** methodology (supporting). **Qianmei Sun:** formal analysis (supporting), investigation (supporting), methodology (supporting). **Zhiyi Gao:** funding acquisition (equal), resources (equal). **Jing Chang:** conceptualization (equal), formal analysis (supporting), investigation (equal), methodology (equal), validation (lead). **Wei Tang:** conceptualization (equal), funding acquisition (lead), investigation (equal), project administration (lead), resources (lead), supervision (lead), writing – review and editing (lead).

## Funding

The project was funded by Beijing Natural Science Foundation (Grant L242041), National High‐Level Personnel of Special Support Program (Grant SQ2024QB00757), National Natural Science Foundation of China Youth Program (Grant 62204246), Zhejiang Provincial Natural Science Foundation Exploration Project (Grant LQ23F040004), Ningbo Municipal Natural Science Foundation General Program (Grant 2023J326), The National Key Research and Development Program of China (Grant 2023YFB2604600) and China Postdoctoral Science Foundation (Grant 2024M753184).

## Conflicts of Interest

The authors declare no conflicts of interest.

## Supporting information

Supplementary Material

## Data Availability

The data that support the findings of this study are available from the corresponding author upon reasonable request.
