# Supplementary material for: Wearable Hybrid Strain–Myoelectric Sensing System for Machine‐Learning‐Assisted Sarcopenia Screening
Source: Small Sci. 2026 Mar 28;6(3):e202500582. doi: 10.1002/smsc.202500582 (PMC13097385; doi:10.1002/smsc.202500582)
Supplement: Supplementary file 1 — Supplementary Material [file SMSC-6-e202500582-s001.pdf]

Supporting information

Wearable Hybrid Strain–Myoelectric Sensing System for Machine-Learning-Assisted Sarcopenia Screening

Ke Wang[1,2,†], Guanbo Min[1,2,†], Tingyu Wang[1,2,†],Chengyu Li[3], En Zhao[1,2], Kun Xu[1,2], Yuer Liang[4], Zhiwei Wang[5], Qianmei Sun[6,*], Zhiyi Gao[7,*], Jing Chang[8,*], Wei Tang[1,2,*]

1 Beijing Institute of Nanoenergy and Nanosystems, Chinese Academy of Sciences, Beijing 101400, China
2 School of Nanoscience and Technology, University of Chinese Academy of Sciences, Beijing 100049, China
3 School of Fashion and Textiles, The Hong Kong Polytechnic University, Kowloon 999077, China
4 Department of Emergency Medicine，Beijing Chaoyang Hospital, Capital Medical University, Beijing, China
5 Department of Orthopaedics, Beijing Chaoyang Hospital, Capital Medical University, Beijing, China
6 Department of Nephrology, Beijing Chaoyang Hospital, Capital Medical University, Beijing, China
7 CAS Key Laboratory of Magnetic Materials and Devices.Ningbo Institute of Materials Technology and Engineering.Chinese Academy of Sciences, Ningbo, 315201, P. R. China.
8 Department of Internal Medicine, Beijing Chaoyang Hospital, Capital Medical University, Beijing, China
† These authors contributed equally to this work.
*e-mail: tangwei@binn.cas.cn, changjing@mail.ccmu.edu.cn, gaozhiyi@nimte.ac.cn, sunqianmei5825@126.com

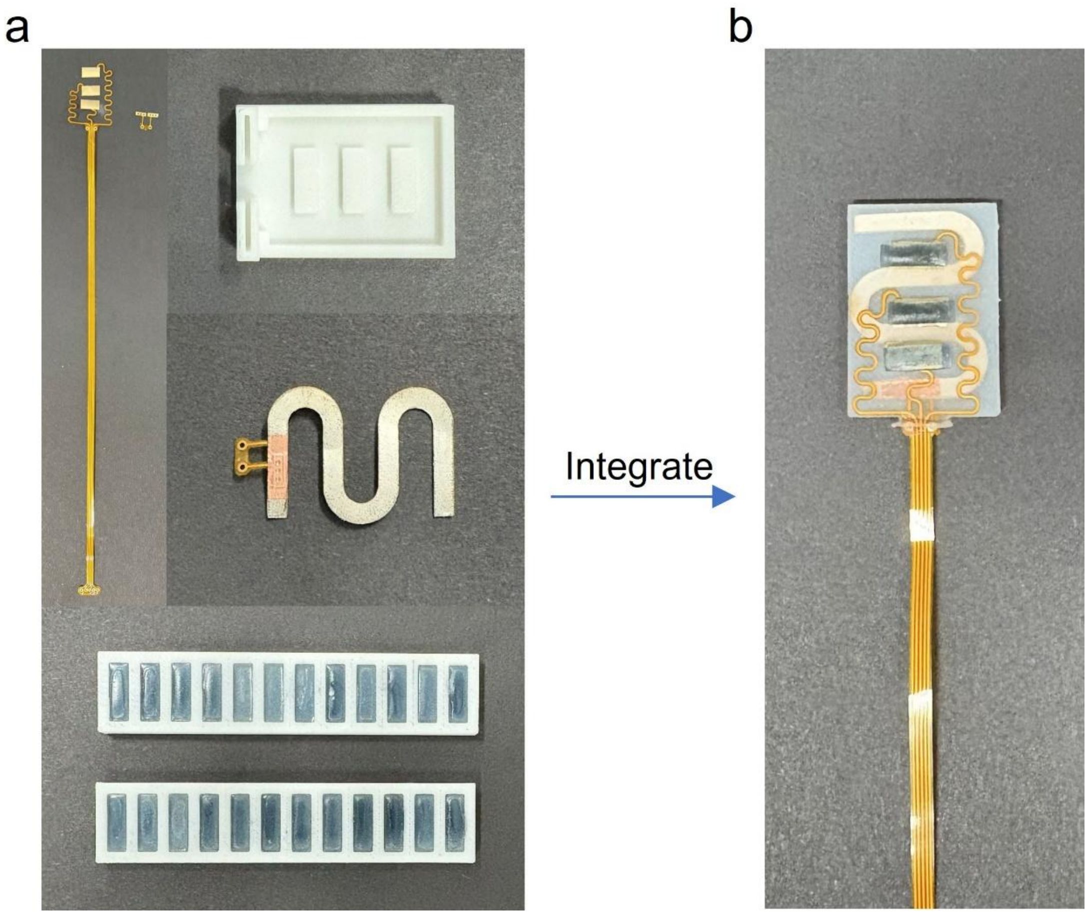

Figure S1. Shown are the detailed components and photographs of the wearable sensor for sarcopenia assessment. (a) The detailed components include FPCB1, FPCB2, a 3D mold, a serpentine piezoelectric PVDF film, and a hydrogel layer[1, 2]. (b) These components are subsequently integrated into a sensor that adheres to the patient's muscle and can simultaneously acquire electromyographic and piezoelectric signals.

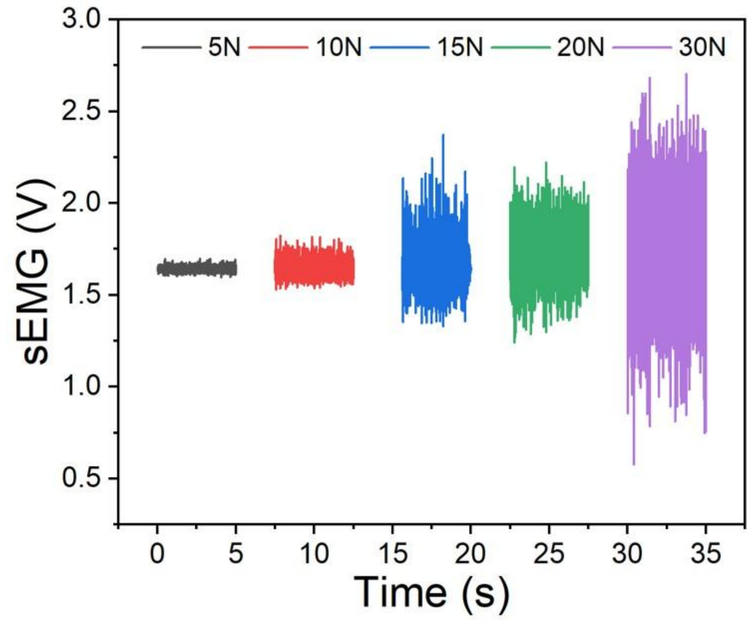

Figure S2. Schematic of electromyographic signals recorded during a standard grip-strength test at various force levels (5 N, 10 N, 15 N, 20 N, and 30 N).

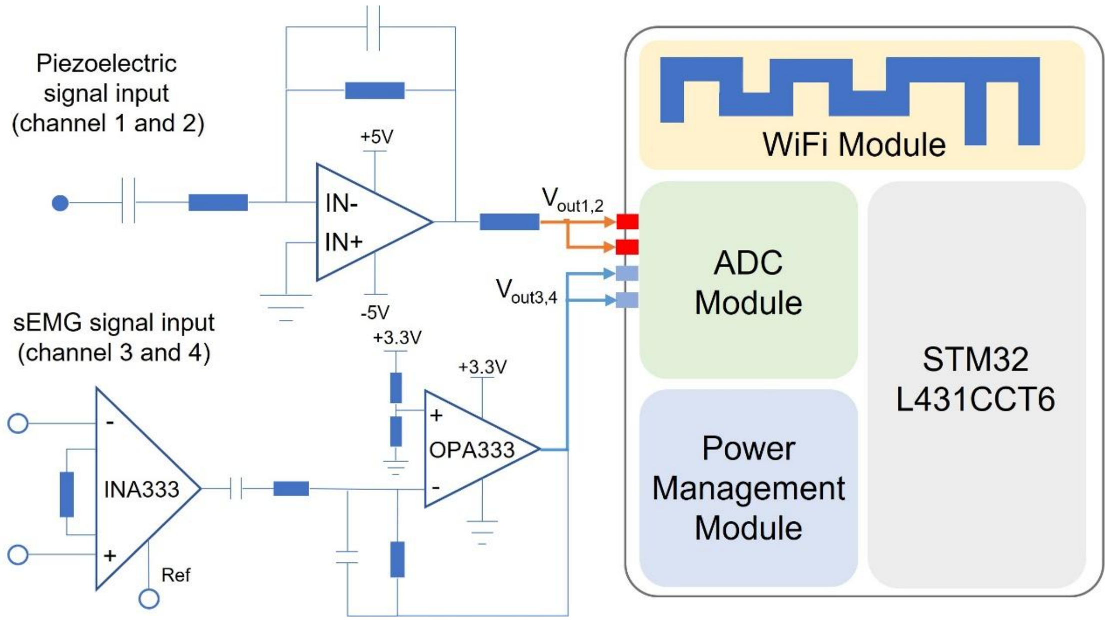

Figure S3. Schematic of the WSAS signal-processing flow. The piezoelectric and electromyographic signals are amplified by their respective amplifier circuits, undergo analog-to-digital conversion and signal processing under the control of the main controller, and are ultimately transmitted wirelessly to the terminal via the Wi-Fi protocol[3].

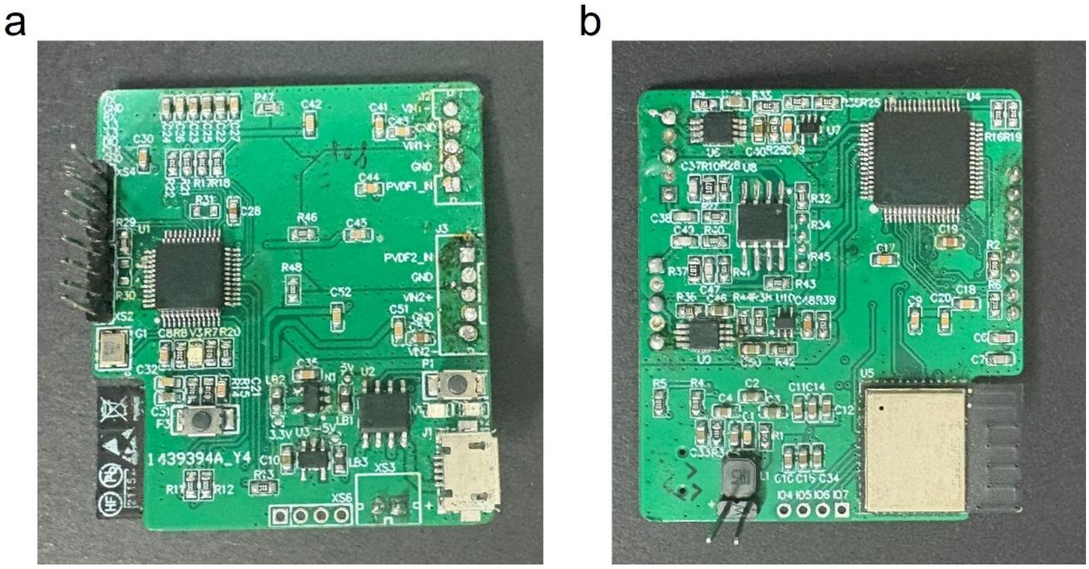

Figure S4. Photograph of the integrated signal-processing circuit, showing the signal amplification, signal conversion, main control unit, and wireless transmission module.

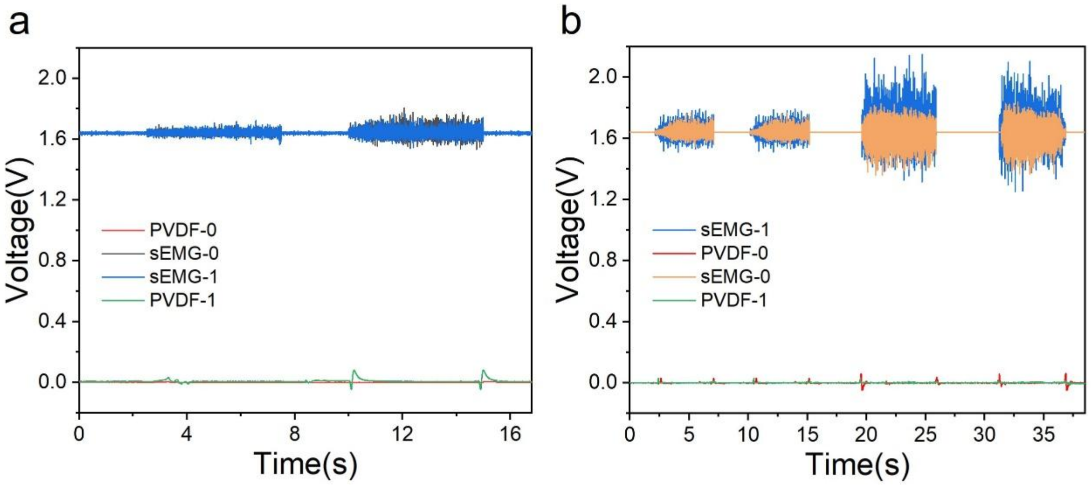

Figure S5. Unnormalized data of the patient before and after being diagnosed with sarcopenia. (a) Unnormalized data corresponding to Fig. 2D(i); (b) Unnormalized data corresponding to Fig. 2D(ii).

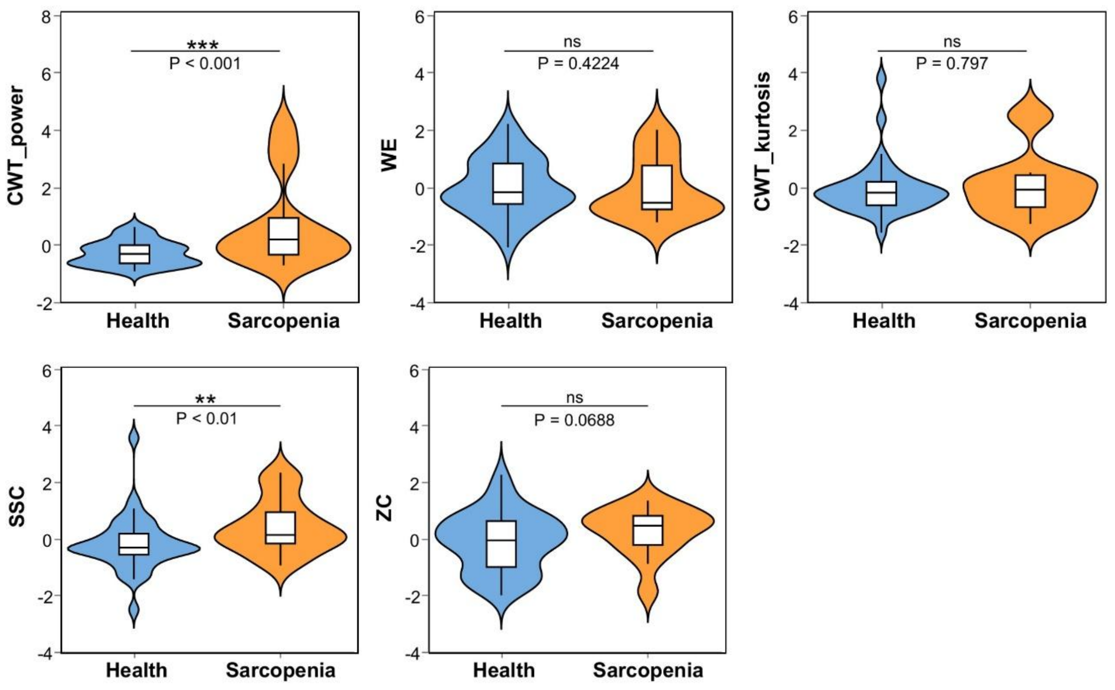

Figure S6. Violin-plot matrix illustrating the differences in CWT_power, WE, CWT_kurtosis, SSC, and ZC. CWT_power differed significantly between the sarcopenia patients and the healthy group, SSC also showed a difference, whereas WE, CWT_kurtosis, and ZC did not exhibit clear differences in this study.

Zero-crossing count (ZC): this feature refers to the number of times the sEMG waveform crosses the zero baseline within a given time window.

$$ZC = \frac{1}{N}\{sgn[x(i)x(i+1)]\}$$

Slope-sign change (SSC): this feature denotes the number of times the sign of the amplitude's rate of change reverses within the sEMG waveform, and it is often used to indicate that the surface EMG signal is about to fluctuate.

$$SSC = \frac{1}{N}\sum_{i=1}^{N-1} f(i)$$

$$f(i) = \begin{cases} 1 \;,\; x(i) > x(i-1), x(i) > x(i+1) \; or \; x(i) < x(i-1), x(i) < x(i+1) \\ 0 \;,\; |x(i) - x(i+1)| > x_{th} \; or \; |x(i) - x(i-1)| > x_{th} \\ else \end{cases}$$

Table S1. Descriptive statistics of all participants represented as mean (±SD).

Total(n=75)	Male(n=47)	Female(n=28)
Age(year)	79.5±11.5	72.6±10.2
Heigth (cm)	171.2±5.9	159.4±4.1
Weight (kg)	68.3±14.6	60.8±8.2
FFM of Right Arm	2.1±0.6	1.6±0.3
FFM of Left Arm	2.0±0.6	1.6±0.3
Arm Muscle Circumference	23.3±3.0	23.1±1.9
SMI	7.9±1.3	6.8±0.7

References

1 Wang S, Yu L, Wang S, et al., Strong, tough, ionic conductive, and freezing-tolerant all-natural hydrogel enabled by cellulose-bentonite coordination interactions. *Nat Commun*. 2022, **13**, 1

2 Gu Y, Xu C, Wang Y, et al., Compressible, anti-fatigue, extreme environment adaptable, and biocompatible supramolecular organohydrogel enabled by lignosulfonate triggered noncovalent network. *Nat Commun*. 2025, **16**, 1

3 Liu D, Zhang D, Sun Z, et al., Active-Matrix Sensing Array Assisted with Machine-Learning Approach for Lumbar Degenerative Disease Diagnosis and Postoperative Assessment. *Adv Funct Mater*. 2022, **32**, 21
